# Essential Oils Prime Epigenetic and Metabolomic Changes in Tomato Defense Against *Fusarium oxysporum*

**DOI:** 10.3389/fpls.2022.804104

**Published:** 2022-03-29

**Authors:** Serine Soudani, César Poza-Carrión, Noelia De la Cruz Gómez, Azucena González-Coloma, María Fé Andrés, Marta Berrocal-Lobo

**Affiliations:** ^1^ETSIMontes, Forestal y del Medio Natural, Departamento de Sistemas y Recursos Naturales, Universidad Politeìcnica de Madrid, Madrid, Spain; ^2^Instituto de Ciencias Agrarias, Consejo Superior de Investigaciones Científicas, Madrid, Spain

**Keywords:** *Fusarium*, tomato, essential oil, plant priming, seed coating, *Artemisia absinthium*, biopesticide, epigenetics

## Abstract

In this work, we studied the direct and indirect plant protection effects of an *Artemisia absinthium* essential oil (AEO) on tomato seedlings against *Fusarium oxysporum* sp. *oxysporum radicis lycopersici* (*Fol*). AEO exhibited a toxic effect *in vitro* against *Fol*. Additionally, tomato seedlings germinated from seeds pretreated with AEO and grown hydroponically were protected against *Fol*. Plant disease symptoms, including, water and fresh weight loss, tissue necrosis, and chlorosis were less pronounced in AEO-treated seedlings. AEO also contributed to plant defenses by increasing callose deposition and the production of reactive oxygen species (ROS) on seed surfaces without affecting seed germination or plant development. The essential oil seed coating also primed a durable tomato seedling defense against the fungus at later stages of plant development. RNA-seq and metabolomic analysis performed on seedlings after 12 days showed that the AEO treatment on seeds induced transcriptomic and metabolic changes. The metabolomic analysis showed an induction of vanillic acid, coumarin, lycopene, oleamide, and an unknown metabolite of m/z 529 in the presence of *Fol*. The *StNRPD2* gene, the second largest component of RNA polymerases IV and V directly involved in *de novo* cytosine methylation by RNA-directed DNA methylation (RdDM), was highly induced in the presence of AEO. The host methionine cycle (MTC) controlling trans-methylation reactions, was also altered by AEO through the high induction of S-adenosyl methionine transferases (SAMts). Our results suggest that AEO treatment could induce *de novo* epigenetic changes in tomato, modulating the speed and extent of its immune response to *Fol*. The EO-seed coating could be a new strategy to prime durable tomato resistance, compatible with other environmentally friendly biopesticides.

## Introduction

Worldwide policies to decrease dependence on toxic pesticides means that new environmentally friendly biopesticides need to be developed. These natural compounds will also help protect the aquatic and agroforestry environment, soil health and biodiversity. According to European Commission policy, the development of new strategies and natural sources of biopesticides is one of the challenges that must be addressed to diminish levels of pesticide residue in food and feed and the corresponding risks for human health (European Commission, 2021; Montanarella and Panagos, 2021). Aromatic plants, studied for their fungistatic, insecticidal, larvicidal, and bactericidal compounds, are currently one of the main sources of natural compounds in the biosphere. The biopesticide potential of the millions of compounds acting directly to protect crops is growing exponentially in tandem with agricultural requirements around the globe (Montanarella and Panagos, 2021).

The plant defense response is accompanied by an extensive transcriptional reprogramming of defense-related genes. The well-known processes which enable systemic acquired resistance (SAR) (Durrant and Dong, 2004), pathogen triggered immunity response (PTI) and enhanced triggered immunity (ETI) (Macho and Zipfel, 2014; Nishimura and Dangl, 2014; Zipfel and Oldroyd, 2017), form part of the priming process in plants. Priming is “the physiological state that enables cells to respond to very low levels of a stimulus in a more rapid and robust manner than non-primed cells.” A transgenerational and non-transgenerational or short term-inheritance of defense-related priming, improving the stability of this priming effect, has recently been shown in plants (Molinier et al., 2006; Pastor et al., 2013; Gully et al., 2019).

Essential oils (EOs) can act as priming molecules both in biotic and abiotic plant stress responses (Bertrand et al., 2021). EOs can be an effective and sustainable tool to control seedborne diseases (Klein et al., 2017; Spadaro et al., 2017). Seed priming prior to sowing is a promising strategy insofar as it makes seed more tolerant to disease and increases the yield and quality of high-value crops such as tomato. Seed priming alleviates stress at germination thus increasing seedling emergence and successful establishment of the seedling. Successful germination determines the vigor of seedling growth at later stages (Acharya et al., 2020). To date, few studies have been conducted on the transcriptomic and metabolomic responses of tomato seeds coated with essential oil to combat phytopathogens or on the assumed “*de novo*” molecular synthesis of immunity-related compounds for priming effect of such treatments (Kaplan et al., 2006; Ben-Jabeur et al., 2015; Rocha et al., 2019).

In this work we studied the transcriptomic and metabolomic responses of tomato seeds coated with an antifungal EO against the phytopathogen *Fusarium oxysporum*. The interaction between tomato and *Fusarium oxysporum f.* sp. *lycopersici* (*Fol*) serves as a model to study the molecular basis of disease resistance and susceptibility. Gene-for-gene interactions in this system have provided the basis for the development of tomato cultivars resistant to *Fusarium* wilt disease. *Fol* is one of the phytopathogens with the greatest impact on the agroforestry environment, producing damping-off disease and reducing tomato (*Solanum lycopersicum*) productivity worldwide (Windels, 2000; Carvalho et al., 2006; Berrocal-Lobo and Molina, 2008; Ma et al., 2013; Edel-Hermann and Lecomte, 2019).

*Artemisia absinthium* is a medicinal plant distributed principally throughout temperate regions in Europe, Asia, and Africa (Bora and Sharma, 2011). *Artemisia* species are current used in industry making them good candidates as a source of new biopesticides to protect plants (Bailen et al., 2013; Kundu et al., 2021). As part of our ongoing project on the sustainable production of biopesticides, thujone-free Spanish populations of *Artemisia absinthium* were domesticated, giving rise to a new plant variety (candial) with *cis*-epoxyocimene, chrysanthenol, chrysanthenyl acetate, *trans*-caryophyllene, and linalool as the main components of its essential oil. (−)-*cis*-Chrysanthenol has been identified as the chemical indicator of the antifungal effects of *A. absinthium* (candial) oil (Arraiza, 2017). However, the indirect effects of this EO against pathogenic fungi (such as plant priming) are still unknown.

Here we describe new metabolites and molecular changes associated with the short- and long-term protection of tomato plants germinated from AEO-coated seeds against the high impact phytopathogenic fungus *Fol*. The effects of the EO are discussed based on metabolomic and RNA-seq analysis. This work proposes new uses of EOs as a source of environmentally friendly biopesticides and biotechnological tools.

## Materials and Methods

### Biological Material

Flowering plants of *Artemisia absintium* var. Candial were harvested in 2019 and their essential oil (AEO) extracted by vapor pressure in a stainless steel semi-industrial plant equipped with two 3,000 L vessels as described (Julio et al., 2015).

Untreated tomato seeds (*Solanum lycopersicum* L., *var. marmande*), were kindly provided by Ramiro Arnedo S.A (La Rioja, Spain). Seeds were dried, stored, and maintained at 4°C until use. *Fusarium oxysporum* sp. *oxysporum radicis lycopersici* (*Fol*) was provided by CECT (# 2715), Valencia, Spain. The fungus was grown on potato dextrose broth medium (PDB) at 28°C for 8 days in darkness as previously described in Berrocal-Lobo et al. (2002). Spores were collected in sterile water, filtered, quantified with a Neubauer chamber and stored in 20% (v/v) glycerol at −80°C until use.

### Fungicidal Activity of *Artemisia absinthium* Essential Oil Against *Fusarium oxysporum* sp. *oxysporum radicis lycopersici* Phytopathogen

The assay to determine AEO fungicidal activity was prepared as previously described with little modification (Bailen et al., 2013). Briefly, the conidial concentration of a potato dextrose agar (PDA) was measured using a Neubauer chamber and diluted to a final concentration of the suspension being determined as 10^5^ spores/ml. Spore susceptibility was tested in a 96-well plate using 100 μl Roswell Park Memorial Institute medium (RPMI) to support spore viability, 80 μl DMSO (1%) was used as a negative control (solvent- and drug-free); 80 μl Amphotericin B (5 μg/ml) as positive control. AEO was tested at different concentrations (5, 50, 500, and 1,000 μg/ml). After 24 h of incubation, antifungal activity was determined by an MTT (Thiazolyl blue tetrazolium bromide staining) assay measuring spore viability, proliferation, and cytotoxicity (Berrocal-Lobo et al., 2009). Menadione 1 mM and 25 μl RPMI medium were added per well incubated for 3 h at 37°C. and then removed prior to the addition of 200 μl isopropanol acid (95% isopropanol with 1 M HCl) After 30 min, the resulting-colored solution was quantified by measuring absorbance at 630 nanometers using a multi-well spectrophotometer and the Gen2.01 program. Data was analyzed with the Stat-graphics Centurion 18 program, using a Variance check (*p* > 0.05) and a non-parametric Kruskal-Wallis test.

The mycelial growth inhibition test was performed in 12-well plates. Ethanol was used as a negative control (2% final concentration). 1,950 μl PDA medium, 10 μl of MTT (5 mg/ml), and 40 μl ethanol were mixed in a sterile Falcon tube, shaken in a vortex, and then poured into each well. AEO stock was prepared at 50 mg/ml and serially diluted (0.1, 0.25, 0.5, and 1 mg/ml). Four replicates were used for each treatment. The plates were covered with aluminum foil and incubated at 27°C for 5 days (Morales-Sánchez et al., 2021). The fungal growth was quantified with the ImageJ program by measuring two perpendicular diameters of the grown area (cm^2^) which was calculated using the formula:


Area=D⁢i⁢a⁢m⁢e⁢t⁢e⁢r⁢x⁢π

Data was analyzed using the Stat graphics Centurion 18 program, using a Variance check (*p* > 0.05) and the non-parametric Kruskal-Wallis test.

### Plant Growth Conditions

Tomato seeds were germinated, and seedlings grown for 12 days in an Aralab chamber (Lisbon, Portugal), at 50% humidity (v/v), temperature of 24°C during the day and 18°C during the night, with a 16-h light/8-h dark photoperiod and light intensity of 150 μE⋅m^–2^ per second for all experiments. Seeds were germinated in sterilized distilled water on paper filter discs in 12 well plates with 12 seeds per well. Plants were irrigated with regular water supply.

### *Absinthium* Essential Oil Seed Coating

A 10 mg/ml AEO solution was prepared in 100% ethanol (Sigma-Aldrich, St. Louis, MO, United States) and serially diluted (1.0 and 0.5 mg/ml) for seed coating. Fresh tomato seeds previously stored for 24 h at 4°C were dipped in the solution one by one and then air dried on sterilized aluminum foil under sterile conditions until use. Control seeds were treated with corresponding 100% ethanol dilutions to AEO dilutions in sterilized water for each assay.

### Plant Inoculations

Coated seeds were kept in constant contact with the fungus in a hydroponic system containing 500 μl of *Fol* (10^6^ spores/ml) in water. Control samples were treated with 500 μl of sterilized distilled water with the corresponding dilution of glycerol. Inoculated plants were placed in the growth chamber (Aralab S.L, Lisbon, Portugal) under the growth conditions specified above. Infection was monitored by measuring the disease parameters specified for each assay.

### Disease Symptoms

Disease symptoms of tomato seedlings (*var. marmande*) were measured considering the different seed germination start and finish times (between 4 and 9 days, respectively) and growth stages. Disease symptoms observed after 7 days were rated as follows: “0” normal seed germination and root emergence; “1” delayed seed germination and root emergence compared to 0; “2” germinated seeds with roots shorter than 2 cm compared to control; “3” germinated seeds with roots shorter than 1 cm; “4” no germination and seeds covered in fungus. Disease symptoms observed after 12 days were rated as follows: “0” no disease symptoms on seedlings; “1” delayed growth observed for shoots and roots with no apparent necrosis or chlorosis; “2” light chlorosis and necrosis on aerial part, including main leaves and shoots; “3” high chlorosis on main leaves and necrosis on shoots; “4” failed seedlings. Trypan blue staining was performed as previously described to detect seedling tissue cell death and necrosis (Lichtenthaler and Wellburn, 1983). Briefly, trypan blue solution is prepared: 10 ml lactic acid (85% w:w), 10 ml phenol (TE balanced buffer, pH 7.5–8.0), 10 ml glycerol (≥99%), 10 ml distilled water, 40 mg trypan blue (final concentration of 10 mg/ml). Seedlings were stained for 20 min and rinsed with 100% ethanol overnight and preserved in 60% glycerol until microscopy observation. Diamino-benzidine (DAB) staining performed as described in the literature was used to stain reactive oxygen species (ROS) production on coated and inoculated seeds (Livak and Schmittgen, 2001). Briefly, seeds and seedlings were placed in ethanol (100%) for 24 h. Tissue was treated with DAB solution (1 mg/ml) for 2 min under vacuum and covered with aluminum foil for 2 h at room temperature. The DAB solution was removed and Ethanol (100%) was added for 2 h before placing in glycerol 60%. DAB staining was performed at 30 min, 1 h, 24 h, 7, and 12 dpi. Callose deposition was measured by aniline blue staining of seedlings. Briefly, seeds or leaves previously treated with ethanol 100% for 24 h were stained in darkness using a 0,1 mg/ml water solution of aniline blue (Sigma-Aldrich, St. Louis, MO, United States) at for 30 min. Tissue was then rinsed with distilled water and placed in 60% glycerol at 4°C and mounted on microscopy slides. DAB and trypan blue were detected by bright light and aniline blue was detected using a DAPI/UV filter by fluorescence microscopy using a stereomicroscope (A292/21 Microscopy iScope IS.3153-PLFi/6 with Fluorescence—IS.3153-PLi/6,nEWF 10x/22, with Plan Fluarex PLFi, 4×, 10×, 20×, 40×, and 100× oil lenses including fluorescence: Blue, Green, UV-DAPI, and Red filters, Microsercon SLU, Madrid, Spain) with a charge-coupled device (CCD) digital cooled camera (A292/21 Euromex 20 MP USB 3.0 with 1 inch CMOS sensor), to obtain digital photos. Image processing and quantification of aniline blue (callose) and DAB signals was performed using ImageJ Software and specific plug-in tools for DAB detection (Schneider et al., 2012).

To measure water content and loss, the fresh and dry weight of seedling tissue was measured, fresh plants were oven-dried at 85°C for 2 days and weighed. The moisture content of individual samples was calculated as follows.


$$\text{WC}=\left(\text{FW}-\frac{\text{DW}}{\text{FW}}\right)\times 100$$

Where WC is the water content of individual plants or seedlings, FW is the fresh weight and DW is dry weight.

Total chlorophyll A, B and carotenoid content was determined according to Lichtenthaler and Wellburn (1983), with little modification. Briefly, 500 mg of fresh leaves were collected in a sterilized Eppendorf with borosilicate glass boils in liquid nitrogen. Samples were then ground in 5 ml of acetone (90%) and centrifuged at 3,000 g for 10 min. The supernatant was collected, and the absorbance of samples was measured using a spectrophotometer (Hach DR 2000, Hach Co., Loveland, CO, United States) at three wavelengths: 662, 644, and 470 nm. Pigment content was then calculated following authors’ specifications and expressed as mg 100 g^–1^, related to fresh weight (fw).


$$C\,a=\left(13,96\times A_{665}\right)-\left(6,88\times A_{650}\right)$$

$$C\,b=\left(24,96\times A_{650}\right)-\left(7,32\times A_{665}\right)$$

$$C\,t=\frac{\left(1000\times A_{470}\right)-\left(2,05\times C\,a\right)-\left(114,8\times C\,b\right)}{245}$$

Where Ca is Chlrolophyl a, Cb is Chlrolophyl b, and Ct is Carotenoids.

### Statistical Analysis

The Stat Graphics Centurion XVI.II program (Stat Point Technologies, Inc., Warrenton, VA, United States) was used for all data analysis relating to plant growth and disease parameters. A one-way analysis of variance (ANOVA) and Duncan’s mean comparison test were performed for all experiments and *t*-tests with a significance level of 0.05%. In the case of non-homogeneous variance, a non-parametric Kruskal–Wallis test was used.

### RNA Quantitative Real-Time-PCR Analyses

Quantitative Reverse Transcription-PCR analysis was performed for RNA-seq data validation. Total RNA was isolated from frozen tomato tissue, separately analyzing roots and aerial parts corresponding to shoots. TRIzol Reagent (Invitrogen^®^, Carlsbad, CA, United States) was used according to the manufacturer’s protocol along with chloroform. RNA samples were then treated with High pure RNA isolation kitto remove trace amounts of genomic DNA (Roche, Manheimm, Germany). RNA samples were analyzed to check quantity using a NanoDrop (UV-Vis ACTG Gene UVS—99. 200 a 850 nm) and quality was checked using Qubit 4.0 (Thermo Fisher Scientific, Madrid, Spain). RNA samples were visualized in 1% agarose gel before next step staining with GelRed (Nippon, Japan). First-strand cDNA synthesis was primed using a hexanucleotide random primer, and cDNA was synthesized using a First-Strand Synthesis Kit (Amersham-Pharmacia, Rainham, United Kingdom) according to the manufacturer’s protocol. A 1.5 μl aliquot of the first-strand synthesis reaction was used as the template for PCR amplification. The program consisted of 3 min at 95°C, 40 cycles of: 30 s at 95°C, 30 s at 60°C, with a final extension step consisting of 7 min at 72°C and dissociation melting curves. The quantitative real-time (qRT-PCR) experiments were performed using a SYBR^®^ Green qPCR master mix (Nzytech, Lisbon, Portugal) with reactions at a final volume of 10 μl per well. Samples were run in a DNA Engine One-Step QRT-PCR machine (Thermo Fisher Scientific, United States). Gene-specific primers were designed using the Primer Express 2.0 program (Applied Biosystems, Foster City, CA, United States), and minimal self-hybridization and dimer formation of primers were determined using the Oligo 6.0 program (Molecular Biology Insights, West Cascade, CO, United States). Primers with annealing temperatures of 58–60°C that amplified products with lengths of about 150 bp were selected and then verified for specificity using a Basic Local Alignment Search Tool (BLAST). The amplification efficiency for each pair of oligonucleotides was calculated as recommended by the manufacturer (Bio-Rad, Hercules, CA, United States) selecting only oligonucleotides with efficiencies above 90% for assays. Gene specific primers used for quantitative real-time PCRs are detailed in Supplementary Table 1. Data was acquired using the One-Step PCR Applied Biosystem Analysis software (Version 2.01), and changes in transcript levels were determined using the 2^–ΔΔ*CT*^ method (Livak and Schmittgen, 2001). Data points were compared using *t*-tests. Three independent biological replicates from different assays were used with three technical replicates in each experiment.

### Construction of RNA-Seq Libraries

Total RNA from three independent biological replicates was extracted as detailed previously. For each sample, 1 μg of total RNA was used to construct the Illumina sequencing libraries according to the manufacturer’s instructions (TruSeq Stranded mRNA LT Sample Prep Kit). Libraries were sequenced using the Illumina HiSeq 2500 platform (Biomarker Technologies) and 150 bp paired-end reads were generated.

### Analysis of RNA-Seq Data

About 4 Gb of high-quality 150-bp paired-end reads were generated from each library and the quality of the clean reads was checked using the Q < 20 threshold. To reduce analysis bias, artifacts such as low-quality reads, adaptor sequence, contaminant DNA, and PCR duplicates were removed using Cutadapt.^1^ Trimmed reads were mapped to the reference genome with HISAT2 splice-aware aligner (Pertea et al., 2016). The tomato reference genome and gene model annotation files were downloaded from the genome website browser (SGN release version SL2.50).^2^ Known genes and transcripts were assembled using String Tie with aligned reads (Kovaka et al., 2019) based on the reference genome model (SL2.50). After assembly, gene/transcript abundance was calculated in the read count and normalized values obtained, i.e., FPKM (Fragments Per Kilobase of transcript per Million mapped reads) and TPM (Transcripts Per Kilobase Million) for each sample using the feature counts function of the Bioconductor (Huber et al., 2015) package R subread (Liao et al., 2019) (strand Specific = 0, is Paired End = TRUE, require Both Ends Mapped = TRUE, primary Only = TRUE, ignore Dup = TRUE). Differentially expressed genes (DEGs) between samples were identified using the DESeq2 package (Love et al., 2014) with standard parameters (fold-change was ≥ 1 and FDR-adjusted *P*-value < 0.05). Average gene expressions in the three biological replicates were used for DEG identification (Supplementary Tables 2, 3).

### Gene Ontology Enrichment Analysis and Kyoto Encyclopedia of Genes and Genomes Pathway Analysis

Panther GO^3^ was used for Gene Ontology (GO) enrichment. The GO enrichment analysis provided all the GO terms which were significantly enriched in the DEGs relative to the genomic background, and DEGs were filtered according to cellular components, molecular functions and biological processes. Kyoto Encyclopedia of Genes and Genomes (KEGG)^4^ is a main pathway-related database. Based on the comparison of the DEGs to the genomic background, pathway enrichment analysis pinpointed the enriched pathways.

### Validation of RNA-Seq by Quantitative Real-Time-PCR

To validate RNA sequencing reading data, 1 μg total RNA was reverse transcribed into cDNA following the previously described protocol for first strand synthesis using oligo (dT) primers. A Quantitative real-time PCR (qRT-PCR) was performed as previously described under the following conditions: 95°C for 10 min, followed by 40 cycles of 95°C for 15 s and 60°C for 30 s. The fluorescence signal was monitored automatically in each cycle. Relative expression levels of specific mRNAs were measured as previously described using the 2^(–ΔΔ*Ct)*^ analysis method (Livak and Schmittgen, 2001), and expression values were normalized using the β-Actin gene. A regression line was calculated to analyze the correlation between Log_2_ RNA-seq readings and quantitative real-time PCR Ct results from 12 independent RNA samples and five genes for each tissue (Supplementary Figure 1). Three independent biological replicates were analyzed for each sample. Primers used in this study are listed in Supplementary Table 1.

### Metabolomic Analysis

#### Tomato Extract Preparation

Treated and untreated tomato seedlings (12 days old, 12 seedlings per replica with three biological replicas) were frozen in liquid N and then extracted with MeOH. Extracts were filtered and kept at −20°C until HPL-MS analysis. For GC-MS, the MeOH extracts were partitioned with dichloromethane (DCM), filtered and the solvent evaporated prior to GC-MS analysis.

#### Gas Chromatography Coupled With Mass Spectrometry Analysis of Essential Oil and Tomato Extracts

The essential oil and DCM fractions of MeOH tomato extracts were analyzed by gas chromatography coupled with mass spectrometry (GCMS) using a Shimadzu GC-2010 gas chromatograph coupled to a Shimadzu GCMS-QP2010 Ultra mass detector (electron ionization, 70 eV). Sample injections (1 μl) were performed using an AOC-20i and equipped with a 30 m × 0.25 mm i.d. capillary column (0.25 μm film thickness) Teknokroma TRB-5 (95%) Dimetil- (5%) diphenylpolisiloxane. Working conditions were as follows: split ratio (20:1), injector temperature 300°C, temperature of the transfer line connected to the mass spectrometer 250°C, initial column temperature 70°C, then heated to 290°C, at 6°C/min intervals. Electron ionization mass spectra and retention data were used to assess the identity of compounds by comparing them with those found in the Wiley 229 and NIST Mass Spectral Database. All extracts (4 μg/μl) were dissolved in 100% DCM for injection. Pure compounds (salicylic acid, chlorogenic acid, and methyl jasmonate from Sigma-Aldrich) were injected and analyzed under the same conditions just described.

#### LCMS Analysis of Tomato Extracts

Methanolic tomato extracts were analyzed by liquid chromatography coupled with mass spectrometry (HPLC-MS) in a Shimadzu apparatus equipped with an LC- 20AD pump and a CTO-10AS VP column oven coupled to a mass spectrometer with a simple quadrupole analyzer LCMS-2020 QP, with an electrospray ionization source (ESI). An ACE 3 C18 column (150 mm × 4.6 mm, 3 μm particle size) with an ACE3 C18 analytical pre-column was used for separation. The compounds were eluted with Methanol (LC-MS grade) (MeOH): MiliQ water with 1% acetic acid 5% MeOH for 5 min, followed by a gradient 5:100% MeOH for 30 min, 100% MeOH for 10 min and 100:5% MeOH for 8 min, with a flow rate of 0.5 ml/min. The nitrogen flow (drying gas for solvent evaporation) was 15 L/min. Electrospray capillary potential was + 4.50 kV and a Full Scan was used in positive mode (m/z 100–700) with a potential of 1.40 kV and a capillary temperature of 250°C. Stock solutions of extracts were injected at 0.25 mg/ml with a 5 μl injection through an automatic injector (SIL-20A XR). All extracts (0.25 μg/μl) were dissolved in 100% MeOH for injection. Pure compounds (lycopene, carotene, salicylic acid, chlorogenic acid, and methyl jasmonate from Sigma-Aldrich) were injected at 0.2 mg/ml and analyzed under the same conditions as described above.

## Results

### *Artemisia absinthium* Essential Oil

Table 1 shows the chemical composition of *A. absinthium* var. Candial (AEO). The oil was characterized by *cis*-epoxyocimene (35%), followed by *cis*- chrysanthenol (9,04%), chrysanthenyl acetate (8,40%), chamazulene (5,01%), and *t*-caryophyllene (4,74%). The composition was like the one previously reported for other crops, highlighting the chemical stability of this plant variety (Julio et al., 2015).

**TABLE 1:** Chemical composition of the *Artemisia absinthium* var. candial essential oil tested.

Compound	Retention time (min)	Area (≥1%)
Linalool	6.451	2.03
(−)-(*Z*)-Epoxyocimene	7.088	34.85
(*E*)-Epoxyocimene	7.303	2.37
Camphor	7.447	1.97
(−)-*cis*-Chrysanthenol	7.765	9.04
Chrysanthenyl Acetate	9.930	8.40
*trans*-Caryophyllene	13.546	4.74
Germacrene-D	14.868	2.41
β-Selinene	14.990	1.45
Dihydrochamazulene	15.520	3.37
Dihydrochamazulene isomer	17.672	1.03
Neointermedeol	18.526	1.20
Chamazulene	19.906	5.01
Geranyl-α-terpinene	24.667	3.30
Geranyl-α-terpinene isomer	24.791	3.24

### Fungicidal Activity of *Artemisia absinthium* Essential Oil Against *Fusarium oxysporum* sp. *oxysporum radicis lycopersici* Phytopathogen

We tested increasing concentrations of AEO (0, 0.005, 0.05, 0.5, and 1 mg/ml) to test spore germination rate. AEO exhibited strong fungicidal activity against *Fol* spores *in vitro* with a significant spore germination inhibition rate of 44.25% ± 1.72, compared to controls at 0.5 mg/ml, with an EC50 of 109.91 as shown in Table 2. Moderate or no fungicidal effects on spore germination were observed at concentrations below 0.005 mg/ml.

**TABLE 2:** *In vitro* analysis of toxic activity of AEO on *Fol* spores.

AEO (μg/ml)	
5	83.58 ± 4.74
50	45.43 ± 0.41
500	44.25 ± 1.72
1000	30.62 ± 2.69
EC_50_	109.91 (63.02–191.68)

*Different concentrations of AEO (μg/ml), were tested on Fol spores (10^5^ spores/ml), by MTT assay (Abs 630 nm).*

### *Artemisia absinthium* Essential Oil Effect on Seed Germination and Tomato Seedling Growth in Presence of *Fusarium oxysporum* sp. *oxysporum radicis lycopersici*

To test for physiological effects of the AEO coating, seeds were treated with 0.5 mg/ml and 1 mg/ml of AEO and germinated in presence (+), or absence (−), of *Fol* (10^6^ spores/ml). Germination rates were determined at 7 dpi, before seed germination was complete, and after 12 days once germination was finished.

**FIGURE 1:**
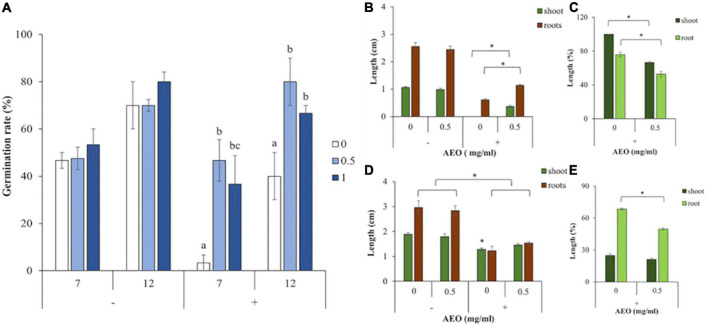
AEO and *Fol* effects on germination rate and growth of tomato seedlings. **(A)** Germination rate measured in non-inoculated (–), or inoculated (+) seedlings, with *Fol* (10^6^ spores/ml), after seed coating with AEO at 0.5 mg/ml (light blue bars), AEO at 1 mg/ml (dark blue bars), or watered controls (white bars), according to section “Materials and Methods”. **(B)** Shoot length (green bars), and root length (brown bars), measured in non-inoculated (–), and inoculated (+) with *Fol* (10^6^ spores/ml), on 7-day-old seedlings, in absence of AEO (“0”), or presence of AEO 0.5 mg/ml (0.5). **(C)** Percent of growth inhibition of shoots (green bars), and roots (brown bars), 7 dpi with *Fol* (+), related to non-inoculated seedlings, in presence of AEO 0.5 mg/ml (0.5) or in watered controls. **(D)** Shoot length (green bars), and root length (brown bars), measured in non-inoculated (–), and inoculated (+) with *Fol* (10^6^ spores/ml), on 12-day-old seedlings, in absence of AEO (“0”), or presence of AEO 0.5 mg/ml (0.5). **(E)** Percent of growth inhibition of shoots (green bars), and roots (brown bars), 12 dpi with *Fol* (+), related to non-inoculated seedlings, in presence of AEO 0.5 mg/ml (0.5) and in watered controls. The * denotes a statistically significant difference using variance check (*P*-value ≤ 0.05) and Duncan test, between bracket samples at **(B,C)**, or (*P*-value ≥ 0.05) and Kruskal-Wallis test at **(D,E)**. The * on **(D)** denotes a statistically significant difference related to all the other treatments.

Figure 1A (−) shows that, in absence of the fungus, there were no significant differences in seed germination rate between AEO treated seeds with 0.5 mg/ml (light blue bars), 1 mg/ml (dark blue bars), or control watered seedlings (white bars).

However, Figure 1A (+), shows that in presence of *Fol* (+), the fungus severely inhibited germination in non-coated seeds (white bars), compared to the AEO treated ones with 0.5 mg/ml (light blue bars) and 1 mg/ml (dark blue bars). This shows that AEO contributed to seedling germination both at 7 and at 12 dpi in presence of *Fol*. Based on these results, the 0.5 mg/ml concentration of AEO, was chosen for further assays.

Seedling root and shoot length was determined after 7 and 12 days in presence (+) and absence (−) of *Fol* (10^6^ spores/ml) from pre-treated seeds with an AEO coating of 0.5 mg/ml (0.5), or 1 mg/ml (1). Figure 1B (−) shows that, at control plants, after 7 days, AEO did not affect shoot growth (dark green bars) or root growth (brown bars), in absence of the fungus (−). However, Figure 1B (+) shows that, in presence of the fungus (+), AEO raises plant tolerance to *Fol* as determined by increased root and shoot length, compared to controls. Figure 1C represents the percent of growth inhibition of shoots (dark green bars) and roots (light green bars) compared to controls in presence of the fungus (+). Growth inhibition of roots and shoots was higher for inoculated plants in absence of AEO.

Figure 1D (−) shows that after 12 days, into control non-inoculated plants (−), AEO did not affect shoot growth (dark green bars) or root growth (brown bars). Furthermore, as shown in Figure 1D, in presence of the fungus (+), an increase in root and shoot length happens, in AEO coated seedlings, showing that AEO contributes to plant tolerate to *Fol*. Figure 1E shows the percent of growth inhibition of shoots (dark green bars) and roots (light green bars) compared to controls in presence of the fungus (+). The inhibition observed on growth, was higher on inoculated plants in absence of AEO, in roots but was not observed in shoots.

### *Artemisia absinthium* Essential Oil Effect on Tomato Seedlings Disease Parameters

**FIGURE 2:**
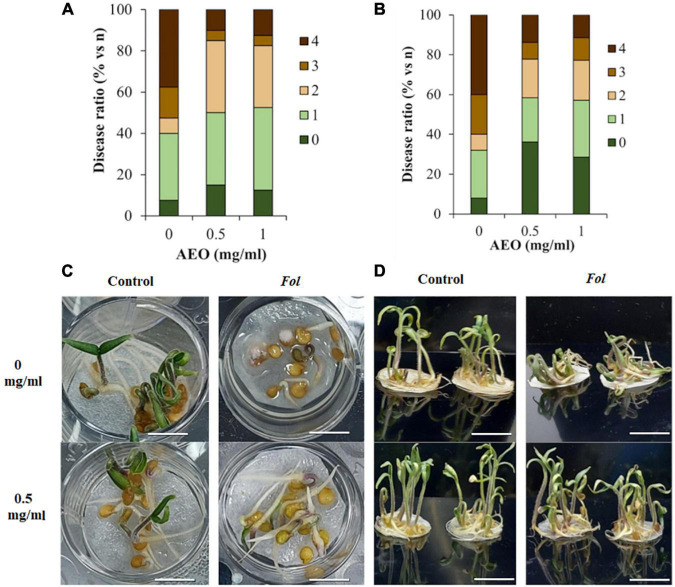
Disease ratio under AEO treatment of seedling inoculated with *Fol*. The effect of AEO (at 0 and 0.5 mg/ml) protecting tomato against *F. solani*. **(A,C)** Disease ratio 7 days after inoculation (+) with *Fol* (10^6^esp/ml), measured as percent of plants with different disease ratios, where: 0: no symptoms and normal germination (shoots and roots longer than 2 cm); 1: Delayed germination, showing radicle with no shoot; 2: Shoot and root measuring less than 2 cm; 3: Necrotic germinated seeds; 4: necrotic ungerminated seeds. **(B,D)** Disease ratio 12 days after inoculation (+) with *Fol* where: 0: no symptoms; 1: growth inhibition of shoots and roots and leaf chlorosis; 2: leaf necrosis and chlorosis; 3: leaf chlorosis and root necrosis; 4: decayed seedlings. Bars: (1 cm).

The disease parameters of the seedlings were studied under hydroponic conditions in the presence and absence of *Fol*. Disease ratios were measured considering the different stages of germination and development of tomato at 7 and 12 days. After 7 days, disease ratios were measured completion of seed germination and after 12 days during heterotrophic growth. A quantitative analysis of disease symptoms indicated that the AEO treatment diminished the disease ratio both at 7 dpi (Figures 2A,C) and 12 dpi (Figures 2B,D).

**FIGURE 3:**
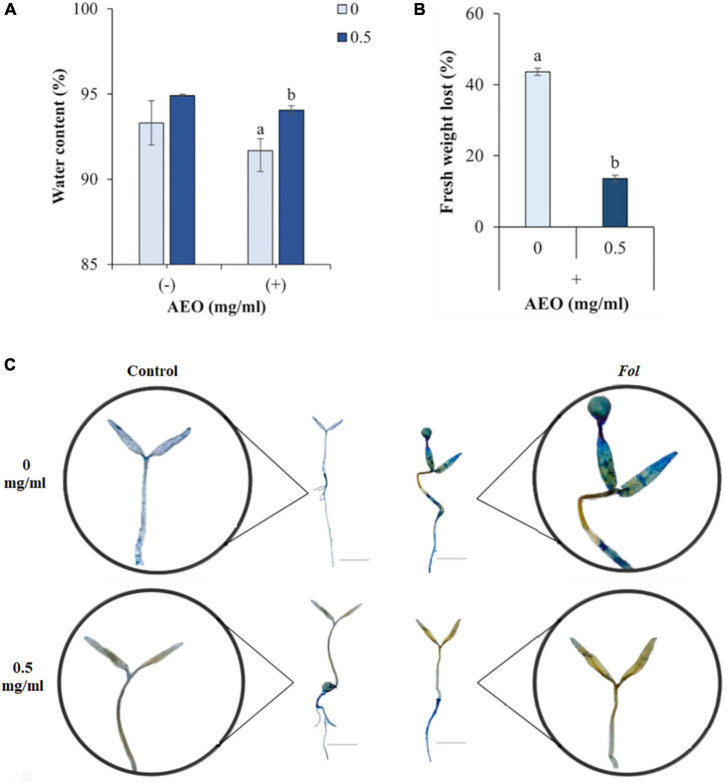
Disease symptoms produced by Fol in AEO treated tomato seedlings. **(A)** Water content (WC), **(B)** Fresh weight loss (FWL), **(C)** Trypan blue (TB) staining showing necrosis induced by *Fol* (10^6^esp/ml). Measurements taken after 12 dpi. Amplification of TB staining of seedlings are shown in circles. AEO treatment at concentrations of 0 (Controls at the top) and 0.5 mg/ml (at the bottom), and in absence (at the left) and in presence (at the right) of *Fol*. Bars (1 cm).

**FIGURE 4:**
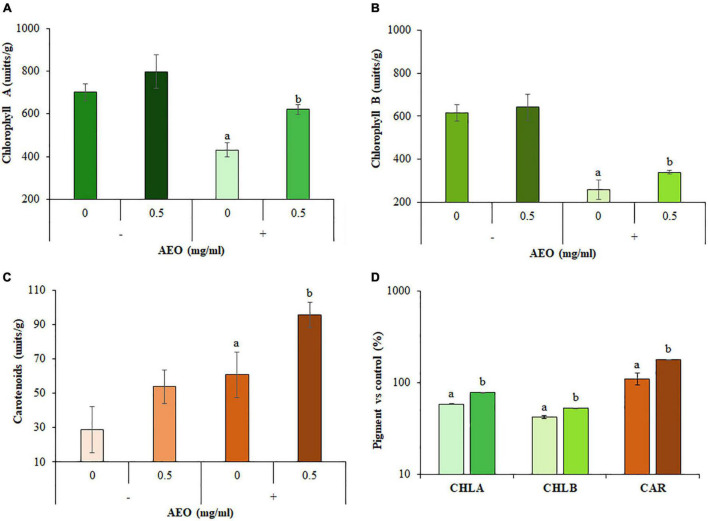
Pigment content of AEO treated seedlings. **(A)** Total content of Chlorophyll A (CHLA). **(B)** Total content of Chlorophyll B (CHLB). **(C)** Total content of carotenoids. **(D)** Pigment increases compared to respective controls. Absorbance (see section “Materials and Methods”) by leave tissue was measured after 12 days on non-inoculated (–) and inoculated (+) seedlings with *Fol* (10^6^ sp/ml), pretreated with AEO at concentrations of 0 (Control) and 0.5 mg/ml. Total pigment content is expressed in (unit/g).

AEO increased plant water content (15%, Figure 3A), and reduced fresh weight loss (FWL) (30%, Figure 3B). AEO also reduced other disease symptoms such as leaf necrosis (Figure 3C) and pigment content measured as total content of chlorophylls A and B (Figures 4A,B), and carotenoids (Figure 4C). Levels of all 3 pigments increased after 12 dpi compared to the corresponding controls into AEO treated seedlings (Figure 4D).

### *Artemisia absinthium* Essential Oil Seed Coating Effect on Reactive Oxygen Species Production and Callose Deposition on Seeds After Germination

**FIGURE 5:**
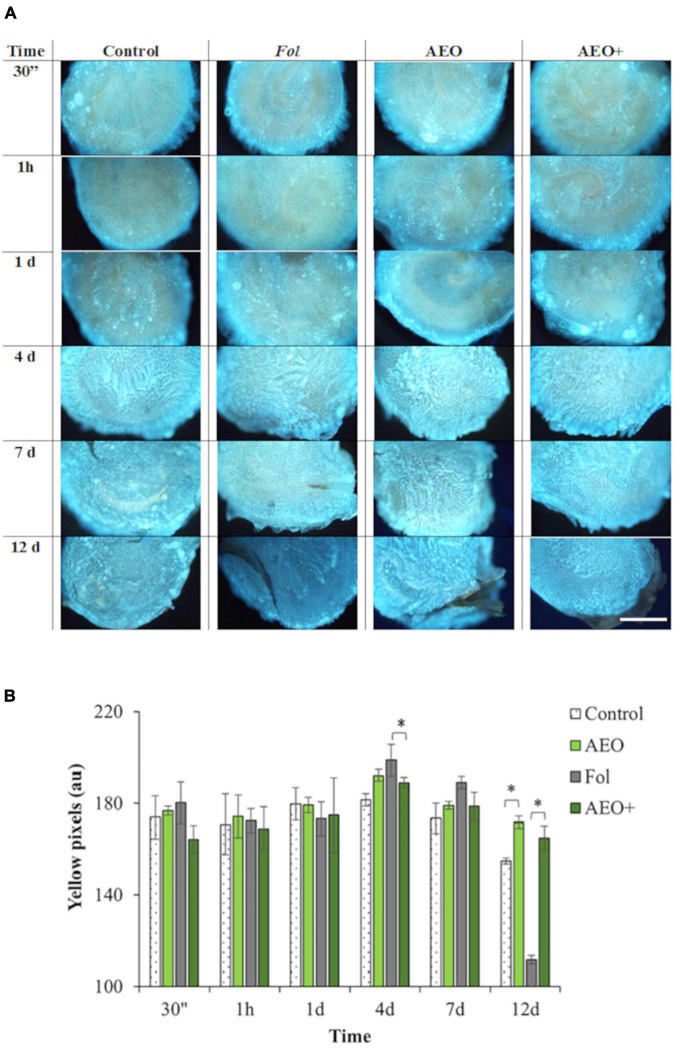
Kinetic of callose deposition on seeds. Kinetic of callose deposition. The * denotes a statistically significant difference using variance check (*P*-value ≥ 0.05) and Kruskal–Wallis test. Tomato seeds were coated with 0.5 mg/ml of AEO. **(A)** Production of callose on seed surfaces was followed by fluorescence staining of callose with aniline blue (see section “Materials and Methods”) after 30 min, 1 h, 24 h, 4, 7, and 12 days (see section “Materials and Methods”), on non-inoculated (–) and inoculated (+) seeds with *Fol* (10^6^ sp/ml), pretreated with AEO concentrations of 0 (Controls) and 0.5 mg/ml. **(B)** Histogram showing quantification of yellow pixels produced by callose measured with Image J program (see section “Materials and Methods”). Bars (1 mm).

**FIGURE 6:**
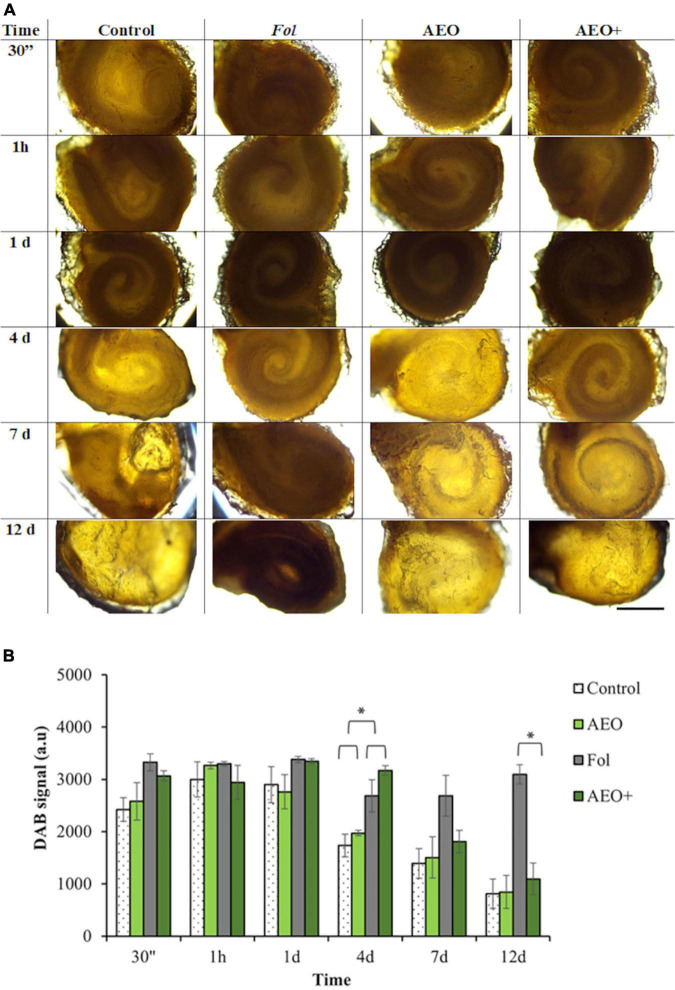
Kinetic of Reactive Oxygen Species Production (ROS) on seeds. In day 4, the * denotes a statistically significant difference using variance check (*P*-value ≤ 0.05) and Duncan test. In day 12, the * denotes a statistically significant difference using variance check (*P*-value ≥ 0.05) and Kruskal–Wallis test. Tomato seeds were coated with 0.5 mg/ml of AEO. **(A)** Production of ROS on seed surfaces was followed by DAB staining of ROS production (see section “Materials and Methods”), after 30 min, 1 h, 24 h, 4, 7, and 12 days, on non-inoculated (–) inoculated (+) seeds with *Fol* (10^6^ sp/ml), pretreated with AEO at concentrations of 0 (Controls) and 0.5 mg/ml. Bars (100 pixel). **(B)** Histogram showing quantification of DAB signal measured with Image J program (see section “Materials and Methods”). Bars (1 mm).

To specifically determine how AEO might protect seeds and seedlings against *Fol*, a kinetic of seed response to AEO treatment (0.5 mg/ml) was performed. Coated seeds were stained with DAB and aniline blue to determine the reactive oxygen production (ROS) of seeds throughout the germination process and the effect of AEO on the callose deposition process, respectively. Staining intensity was measured after 30 min, 1 h and at 1, 4, 7, and 12 days after the coating treatment in the presence and absence of Fol, AEO or both treatments. As shown in Figure 5A, the AEO coating treatment increased callose deposition during the germination period, between days 4 and 7. Callose quantification (see section “Materials and Methods”), confirmed the observed increase in callose deposition. The increase in callose was maintained up to 12 days in the AEO treated, inoculated and non-inoculated seedlings (Figure 5B). A high level of ROS production was also observed before germination in all seeds, as was a reduction in ROS production during seed germination (between 4 and 7 days) in control and AEO treated seeds in absence of the fungus (Figure 6A). The ROS level was high, on infected with *Fol*, even after the germination state. However, ROS levels in control seeds, seeds treated with AEO and AEO pre-treated and infected seeds, decreased once the germination process started (Figure 6A). The quantification of DAB confirmed those data (Figure 6B). A similar staining analysis was performed on seedling leaves but no ROS or callose depositions were observed in that tissue (data not shown).

### Analysis of Seedling Response to *Artemisia absinthium* Essential Oil by RNA-Seq and Metabolomics

**FIGURE 7:**
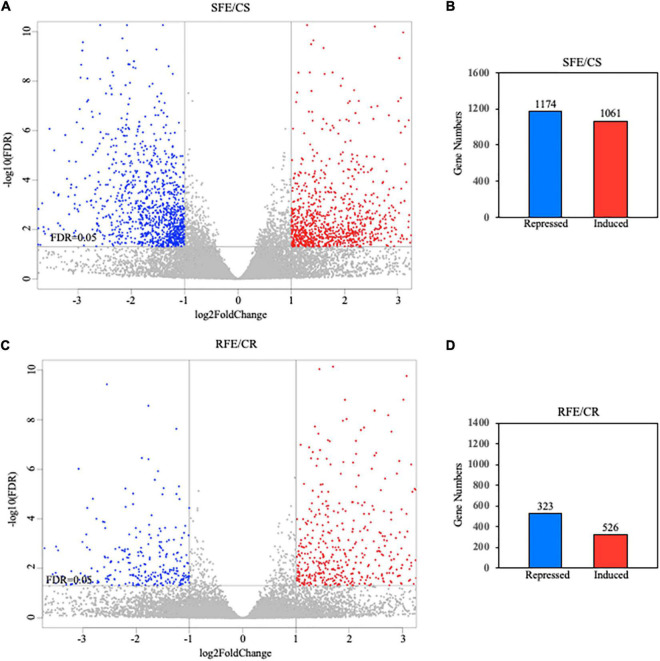
Comparison of RNA-seq data between shoot and root assays. **(A,C)** Volcano plots made on shoots (upper) and roots (lower). Up-and downregulated DEGs are reported as red and blue dots, respectively. No DEG contribution represented as gray dots. **(B,D)** Number of induced or transcriptionally repressed genes corresponding to volcano analysis.

**FIGURE 8:**
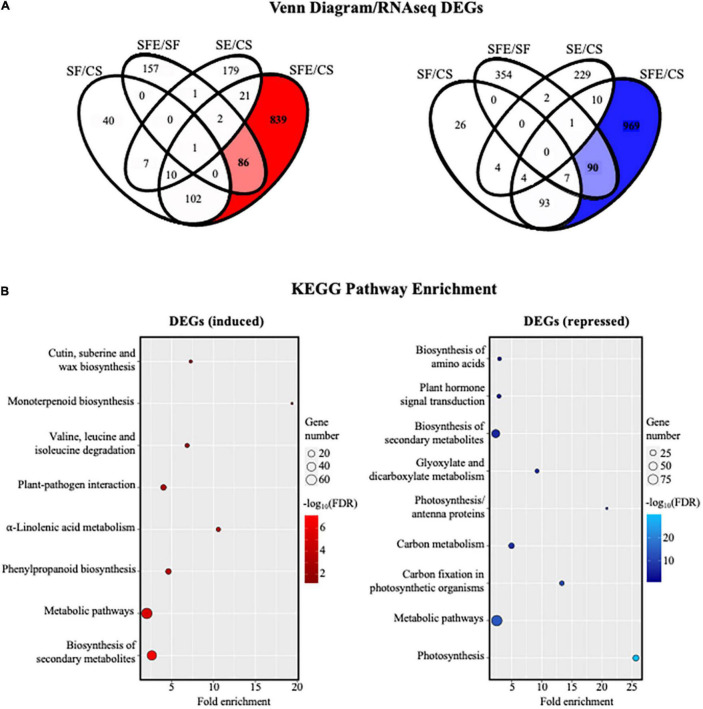
Analyses of RNA-seq data from seedlings (shoots). **(A)** Identification of differentially expressed genes (DEGs) between different types of treatments using Venn diagram software (VENNY ^2.1^; https://bioinfogp.cnb.csic.es/tools/venny/index.html). Correspondences are (CS, control plants; SE, plants treated with AEO 0.5 mg/ml; SF, seeds inoculated with *Fol* 10^6^ sp/ml; and SFE, AEO coated seeds 0.5 mg/ml inoculated with *Fol*. **(B)** KEGG pathway enrichment analysis of DEGs obtained from Venn diagram in RNA-seq. Gene number corresponds to the number of target genes in each pathway. Fold enrichment is the ratio (observed/expected) of the number of DEGs in a certain pathway. FDR-value is corrected *P*-value. The color and size of the dots represents the range of the –log_10_(FDR)-value and the number of DEGs mapped to the indicated pathways, respectively. Top enriched pathways are shown in the figure.

While the short-term compounds involved in tomato’s recognition of *Fol* have been described, (Kaplan et al., 2006; Berrocal-Lobo and Molina, 2008; de Lamo and Takken, 2020) studies characterizing the long-term and “*de novo*” synthetized molecular compounds involved in plant tomato defense against *Fol* are still scarce. This work focuses on characterizing the effect that AEO has on tomato’s long-term defense response to *Fol*. RNA-seq sequencing of plant genome after 12 days of inoculation with the fungus enabled us to determine the longer-term transcriptional changes produced by AEO in terms of the immunity response of tomato seedlings (see section “Materials and Methods”). A volcano Plot performed based on DEG analysis allowed us to determine that the number of genes transcriptionally upregulated or downregulated was substantially higher in shoots than in roots, 1,061 being induced in shoots compared to 526 in roots, 1,174 genes repressed in shoots compared to 323 in roots (Figures 7A–D). DEG analysis of shoot tissue showed a significant number of genes that are transcriptionally induced or repressed by the AEO treatment, 925 genes upregulated and 1,059 repressed, respectively (Figure 8A). A small number of genes was sufficient to perform a DEG analysis of the roots. The KEGG analysis determining pathway enrichment (see section “Materials and Methods”) enabled us to identify the metabolomic pathways involved in the biosynthesis of secondary metabolites, linolenic acid, phenylpropanoids, monoterpenoids (McGarvey and Croteau, 1995), amino acid degradation, and plant-pathogen interactions, i.e., the main pathways induced as part of the long-term defense response of tomato seedlings (Figure 8B, left panel). Significant inhibition of other metabolic pathways was repressed, including the biosynthesis of secondary metabolites, the primary metabolism of carbon and glyoxylate and dicarboxylate metabolism, showing a transcriptional plant response to AEO affecting some specific metabolic pathways but not others (Figure 8B, right panel). Raw data were submitted, *GEO accession number GSE186754.*

### Metabolomic Effects of *Artemisia absinthium* Essential Oil on Tomato Seedlings

**FIGURE 9:**
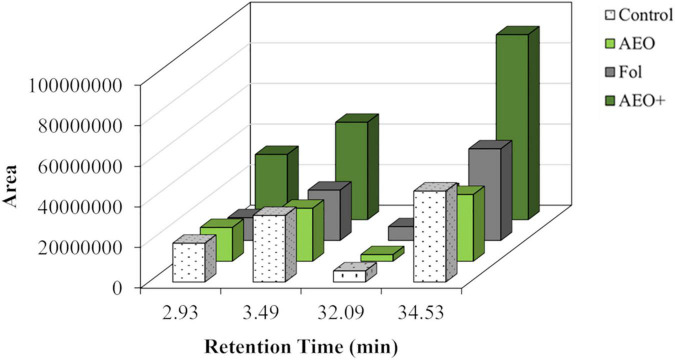
Metabolomic analysis in AEO tomato seedlings. Metabolite induction is represented by arbitrary units. Correspondences are (CS, control plants; SE, plants treated with AEO 0.5 mg/ml; SF, seeds inoculated with *Fol* 10^6^ sp/ml; and SFE, AEO coated seeds 0.5 mg/ml, inoculated with *Fol*).

**FIGURE 10:**
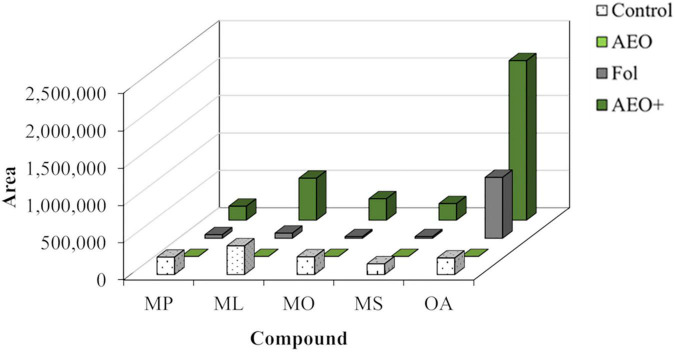
GC-MS analysis of the DCM-soluble fraction of AEO tomato seedlings. The analyzed compounds are palmitic acid methyl ester (MP), linoleic acid methyl ester (ML), oleic acid methyl ester (MO), stearic acid methyl ester (MS), and oleoamide (OA). CS, corresponds to control plants; SE, plants treated with AEO 0.5 mg/ml; SF, seeds inoculated with *Fol* 10^6^ sp/ml; and (SFE, AEO coated seeds 0.5 mg/ml, inoculated with *Fol*).

The mass ions of 12-day-old tomato seedlings analyzed by LC-MS showed induced metabolites of mass ions compatible with vanillic acid (179, M+H), coumarin (147, M+H), lycopene (537, M+H), and an unknown metabolite of m/z 529 that could be related to lycopene (Table 3). All these compounds were induced in the presence of AEO and *Fol*, with the compound of m/z (529) giving the strongest response (Figure 9). GCMS analysis of the dichloromethane fraction of the MeOH extracts (Figure 10) showed a strong induction of the lipid oleoamide also in the presence of AEO plus *Fol*.

**TABLE 3:** LC-MS m/z adducts of main metabolites detected in tomato seedlings extracts analyzed by LC-MS.

Retention time (min)	[M+H]+ m/z	Identification
2.93	179	Vanillic acid
3.49	147	Coumarin
32.10	537	Lycopene
34.53	529	Lycopene-related

## Discussion

In this work we have demonstrated the antifungal effects of *A. absinthium* var. candial vapor pressure essential oil (AEO) against *Fusarium oxysporum* conidia. Previous reports have shown that *A. absinthium* var. candial collected in previous years (2008–2013), was moderately antifungal against the mycelium of *Fusarium* species (*F. moniliforme and F. solani*), with stronger effects against *Botrytis cinerea*. Fractionation of the AEO resulted in the identification of (−)-*cis-*chrysanthenol as the main antifungal compound, followed by linalool (Julio et al., 2015).

The coating of tomato seeds with a vapor pressure essential oil from *A. absinthium* var. candial (AEO) protected seed germination and seedling growth against *Fol*. Our results indicate that AEO protected seeds by directly affecting the fungus but also by the induction of a long-term response in terms of ROS production and callose deposition after germination. These effects were not detected in untreated seeds, where callose and ROS production increased during germination and decreased in the absence of the fungus. There are previous results describing effects of coated seed priming on tomato (Król et al., 2015; Gonçalves et al., 2021).

A long-term response from AEO seed treatment was detected, which maintained higher levels of ROS and callose up to 12 days. Few studies describe callose deposition on tomato seed in the presence of phytopathogens (Zhang et al., 2015; Crespo et al., 2017) and this is the first work to date that describes callose deposition on tomato seeds induced by *Fol* and an essential oil. Our results indicate that AEO might penetrate the seeds once the testa is broken inducing molecular modifications in seeds and undifferentiated embryonic cells, contributing to the long-term tolerance observed in seedlings after 12 days. Our RNA-seq results suggest that the AEO coating treatment might induce specific “*de novo*” molecular changes detected at transcriptional level after 12 days in contact with the fungus. However, the molecular mechanism that might be involved in these effects remain unknown.

This work measures several of the hormones involved in the activation of the short-term signaling pathways related to tomato plant defense (Hernández-Aparicio et al., 2021) such as salicylic acid, jasmonate, methyl-jasmonate, and ethylene. However, the signal levels were not changed significantly compared to controls (data not shown), indicating that they are not the principal molecules involved in the long-term tolerance of tomato seedlings responding to *Fol*. The RNA-seq analysis showed that some other genes related to plant defense responses might also contribute to this tolerance, including genes involved in fatty acid metabolism, peroxidases, terpene synthases (Sun et al., 2016; Zhou and Pichersky, 2020), methyltransferases, and enzymes involved in gene silencing. Transcriptomic analysis showed enriched transcriptional induction on specific secondary metabolism pathways. The terpene synthases were transcriptionally induced. Lycopene and carotenoid synthesis was also transcriptionally induced by AEO in our DEG analysis as previously described for wheat (Colasuonno et al., 2017).

The metabolomic analysis showed that the coating of tomato seeds with a vapor pressure essential oil from *A. absinthium* var. *candial* (AEO) induced several metabolites in tomato seedlings when infected with *Fol*, including the polar compounds vanillic acid and coumarin and the apolar ones lycopene, a metabolite of M+ 528, and the fatty acid derivative oleamide, these latter two being the most highly induced compounds. These results agree with the transcriptional induction of carotenoids and fatty acid metabolic pathways detected by RNA-seq analysis, and the increase in total chlrolophyl A and B and carotenoids found in the pigment analysis. These results might suggest that AEO treatment induces *de novo* changes that remained in the presence of the fungus for long-term plant tolerance.

Vanillic acid is a phenolic allelochemical reportedly present in tomato plants (Méndez and Brown, 2011) which significantly improves salinity tolerance and plant growth performance when externally applied to tomato seedlings (Parvin et al., 2020). Coumarin is an antioxidant, antimicrobial, and growth promoter in plants (Saleh et al., 2015; Nduche et al., 2019; Santra and Banerjee, 2020) and also mitigates salt stress in tomato plants (Parvin et al., 2020). Lycopene is a red carotenoid pigment of M+ (536) found in fruits and vegetables, including tomatoes (Stahl and Sies, 1996). Carotenoids are involved in photosynthesis and photoprotection in plants (Tao et al., 2007). Lycopene has an antimicrobial effect against bacteria and fungi such as *Candida albicans* by inducing apoptosis via ROS production and mitochondrial dysfunction (Choi and Lee, 2015). However, this is the first report on the induction of an apolar compound of M+ (528) in tomato plants treated with AEO and *Fol*. Oleamide (an oleic acid derivative) content increased with UV-B2 treatment in an olive cultivar (Celeste Dias et al., 2018). The role played by oleamide in plants remains unclear, but could be involved in growth/development regulation, stress response, and pathogen interactions (Kim et al., 2013) as shown here.

Additional assays are needed to determine the specific genetic modifications that enable the transcriptional and metabolic changes responsible for the long-term tolerance of tomato observed in this work. The possibility that epigenetic modifications are taking place in seeds resulting in “*de novo*” molecular modification for long-term tolerance of aerial parts opens new perspectives for the use of priming tools. The RNAseq analysis, also detected an inhibition of genes related to redox stress associated to ROS production during photosynthetic electron transport in the chloroplast. This response probably relates chloroplast stress with immunity response to *Fol*. Similarly, mitochondrial stress induced plant resistance, through chromatin changes, against phytopathogenic fungi and bacteria in Arabidopsis (López Sánchez et al., 2021). Therefore the stress produced in organelles involved into primary metabolism, might contribute to plant immunity. Furthermore, the interactions between metabolomics, transcriptomics, redox regulation, and epigenetics as shown here, are under current study in other plant systems (Shen et al., 2016). Future assays will be necessary to dilucidated our hypotheses. Our RNA-seq results, confirmed by quantitative real time PCR, show that *NRPD2* (López et al., 2011) mediating in *de novo* cytosine methylation by RNA-directed DNA methylation pathway (RdDM), is highly induced after 12 days of treatment with AEO in *Fol* infected seedlings (Supplementary Data). *NRPD2* was involved in efficient immunity response to *Botrytis cinerea* (López et al., 2011) and *Pseudomonas syringae pv tomato DC3000* in *Arabidopsis thaliana* (Zhang et al., 2021), and was necessary for reactive oxygen species (ROS) production, and activation of jasmonic acid and salicylic acid signaling pathways. Alterations in chromatin structure were necessary for efficient resistance to that fungus (Walley et al., 2008). To our knowledge, this is the first report relating NRPD2 to tomato immunity response regulation to *Fol*. *NRPD2* was also highly induced at transcriptional level by cucumber mosaic virus (CMV) in *Arabidopsis* being essential for plant immunity and demonstrating the importance of the induction of that gene during plant defense response (Kanazawa et al., 2011). In line with our results, a previous work demonstrated in *Arabidopsis* that DNA methylation is involved in immunity against *Fol* (Le et al., 2014) since RdDM-related mutants showed enhanced susceptibility to this fungus, suggesting that *de novo* methylation process contributes to *Fol* immunity. In addition, in this work the WRKY33 transcription factor, involved in the epigenetic control of plant defense against necrotrophs (Espinas et al., 2016; Ramirez-Prado et al., 2018; Alvarez-Venegas et al., 2019), was also induced specifically by AEO in presence of *Fol* in this work (Supplementary Data). This transcription factor showed increased levels on H3K4me3 on its promoter during *Botrytis cinerea* tomato infection (Crespo-Salvador et al., 2018). We also observed a significant increase in two S-adenosyl-L-methionine-dependent methyltransferases, one induced by real time PCR more than one hundred times (Supplementary Data), on AEO infected tomato seedlings after 12 days of treatment. *S-adenosylmethionine transferases* (SAMts) are responsible for maintaining the plant’s methionine cycle (MTC) in the plant (Mäkinen and De, 2019). MTC connects ethylene and methylation pathways, where SAMs are DNA cytosine methylation markers for transcriptional RNA silencing (RGS), (Pooggin, 2013). The overexpression of SAMs in tomato increased tolerance to ROS stress (Gong et al., 2016) and prevented ROS accumulation in Arabidopsis (Jang et al., 2012). Our results detected very high levels of 1-aminocyclopropane-1-carboxylate oxidase 2 (ACCO), specifically involved in MTC cycle, for the synthesis of ethylene.

Considering that our RNA sequencing results indicate that MTC cycle is altered by AEO primed tomato plants in presence of *Fol*, a molecular analysis including histone epigenetic marks and effects on the offspring will be necessary to characterize this *de novo* long-term tolerance, produced by *A. absinthium* AEO.

## Conclusion

This work demonstrates that the essential oil from *Artemisia absinthium* var. candial primed tolerance in tomato seedlings against phytopathogens such as *Fusarium oxysporum* sp., protecting not only seed germination, but also seedling growth and producing long term effects on plant tolerance after germination by inducing metabolomic and transcriptomic changes. Therefore, this work demonstrates that seed priming might be a useful tool to induce “*de novo*” non-transgenerational epigenetic changes in crops, modulating later responses of aerial parts of the plant, allowing crops to grow and improve tolerance against high impact phytopathogens.

## Data Availability Statement

The datasets presented in this study can be found in online repositories. The names of the repository/repositories and accession number(s) can be found below: NCBI (accession: GSE186754).

## Author Contributions

SS performed all the assays characterizing the physiological response of tomato seeds and seedlings to AEO and *Fol* including sampling for RNA-seq and metabolomic analysis. CP-C designed and carried out the RNA-seq analysis, including DEG, KEG, primer design, and reposition of public data. ND performed RNA extractions and QRT-PCR analysis for RNA-seq confirmation. AG-C and MA performed AEO isolation and chemical characterization and metabolomic analysis and metabolite chemical characterization. MB-L coordinated this work and designed the experiments to characterize tomato responses to AEO and *Fol* and drafted the manuscript. All authors contributed to critical reading and writing of the manuscript and drew up their corresponding figures or tables.

## Conflict of Interest

The authors declare that the research was conducted in the absence of any commercial or financial relationships that could be construed as a potential conflict of interest.

## Publisher’s Note

All claims expressed in this article are solely those of the authors and do not necessarily represent those of their affiliated organizations, or those of the publisher, the editors and the reviewers. Any product that may be evaluated in this article, or claim that may be made by its manufacturer, is not guaranteed or endorsed by the publisher.

## Footnotes

1
https://pypi.org/project/cutadapt/

2
https://solgenomics.net

3
http://www.pantherdb.org

4
http://www.genome.jp/kegg/

## Supplementary Material

Supplementary Table 2 List of selected genes induced by AEO in presence of the fungus.

Supplementary Table 3 List of selected genes repressed by AEO in presence of the fungus.
