# Corrigendum: Essential Oils prime epigenetic and metabolomic changes in tomato defense against *Fusarium oxysporum*

**DOI:** 10.3389/fpls.2024.1443732

**Published:** 2024-06-24

**Authors:** Serine Soudani, César Poza-Carrión, Noelia De la Cruz Gómez, Azucena González-Coloma, María Fé Andrés, Marta Berrocal-Lobo

**Affiliations:** ^1^ ETSIMontes, Forestal y del Medio Natural, Departamento de Sistemas y Recursos Naturales, Universidad Politécnica de Madrid, Madrid, Spain; ^2^ Instituto de Ciencias Agrarias, Consejo Superior de Investigaciones Científicas (CSIC), Madrid, Spain

**Keywords:** *Fusarium*, tomato, essential oil, plant priming, seed coating, *Artemisia absinthium*, biopesticide, epigenetics

In the published article, there was an error in affiliation 1. Instead of “School of Forestry Engineering and Natural Resources, Department of Systems and Natural Resources, Polytechnical University of Madrid, Spain”, it should be “ETSIMontes, Forestal y del Medio Natural, Departamento de Sistemas y Recursos Naturales, Universidad Politécnica de Madrid, Madrid, Spain”.

The authors apologize for this error and state that this does not change the scientific conclusions of the article in any way. The original article has been updated.

