# Supplementary material for: Essential Oils Prime Epigenetic and Metabolomic Changes in Tomato Defense Against Fusarium oxysporum
Source: Front Plant Sci. 2022 Mar 29;13:804104. doi: 10.3389/fpls.2022.804104 (PMC9002333; doi:10.3389/fpls.2022.804104)
Supplement: Supplementary file 1 [file Table_1.docx]

**Supplemental Table 1.** DNA Primers used in this study.

| Gene_ID Primer sequence (5´-3´; forward/reverse) |
| --- |

Solyc04g054380.1 GACATGCACTTATGCAATCTCTTC/CGTTGCTTGATCCACATTCTTC

Solyc04g077490.3 CCTAGTGGACTGGAATGTGTAG/CTCTGACCAAATGTGTCAATGG

Solyc04g005250.3 GATGCTCTTTTGACATTCAAGCAGG/AGCACACATGAAATCTATCAACTCCG

Solyc08g076930.1 AGAAGTTACCGACACCGAATG/CCAAATCGGGCTGGAACTATAC

Solyc05g051200.1 ATTGGAGTTAGAAAGAGGCCAT/CTCATTGATAATGCGGCTTG

Solyc09g066360.1 TGCTGAAGGATCATCGCAAG/ACCTAGCCATACACGAACACC

Solyc11g005330.2 GAAGTTATTACCATTGGTGCTGAGA/TGCAGCTTCCATACCAATCATG

Solyc05g054380.2 GAAGGGGATCCATTGGGACAA/TTCCCATAGCACTATCTTTTCCA

Solyc01g101180.3 ACACCGATGCCATACAGAGG/TCTCCCATCCTTTGACAACTCC

Solyc03g020010.1 CCCATTGACATTCACACCCG/GTTTCCACACCAGGGTTTCC

Solyc11g071760.2 GCTAAGGAGATGCATGAAGGC/CAAACCCTAACAACCCGTCC

Solyc08g075940.3 AAACTAAGACGTAAGCGCCG/ AGAGGGCGCATAATCCTTCC

Solyc04g040180.3. GATGTTGGTACTGGTAGTGGC/ GAACATTTGGAACCTTTGCTGC

Solyc09g014990.3 AGTAGGCCTGCTACTTCTTCC/ ATGGTTGACAGTAATGCACCG
