# Supplementary figures and images for: Essential Oils Prime Epigenetic and Metabolomic Changes in Tomato Defense Against Fusarium oxysporum
Source: Front Plant Sci. 2022 Mar 29;13:804104. doi: 10.3389/fpls.2022.804104 (PMC9002333; doi:10.3389/fpls.2022.804104)

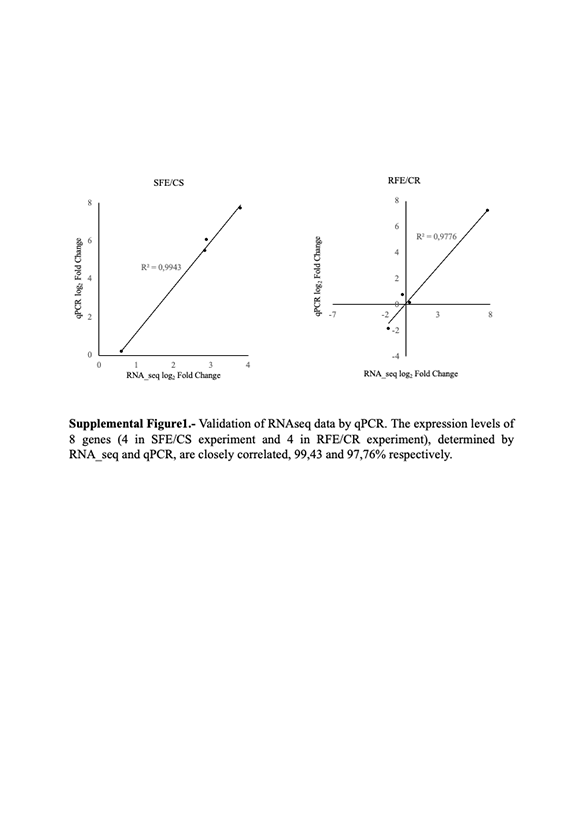

Supplement: Supplementary file 4 [file Image_1.TIFF]

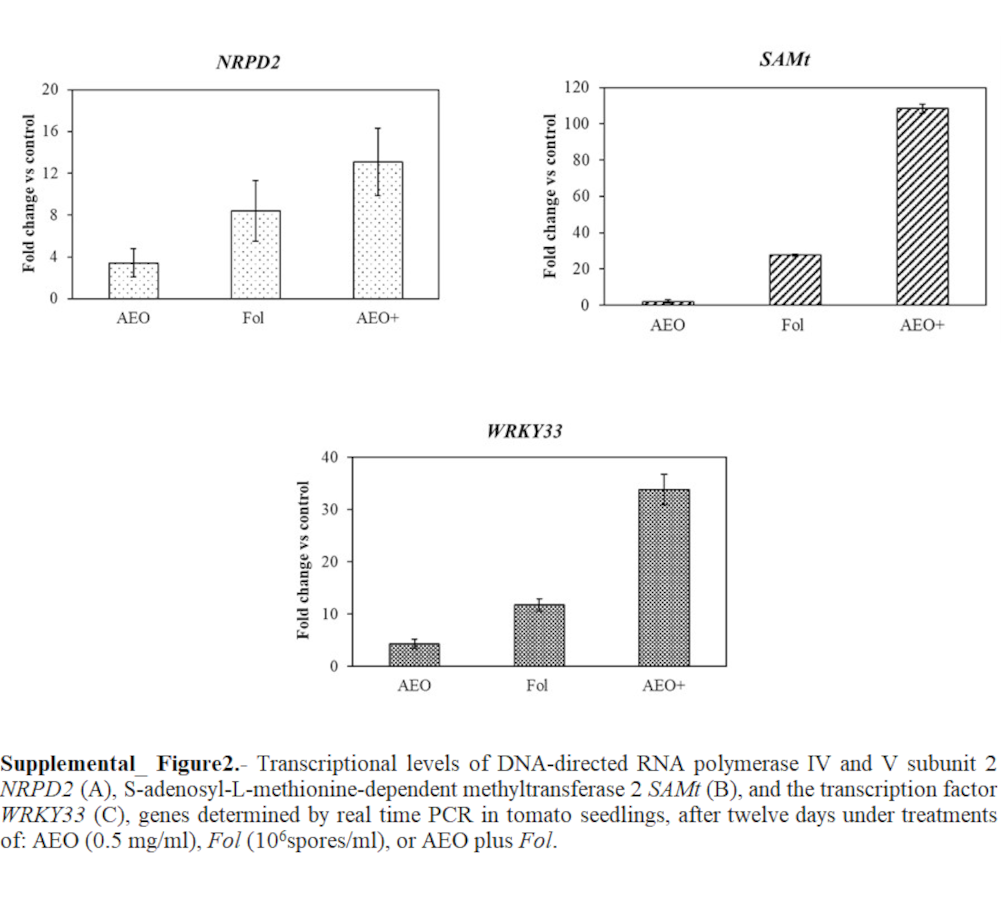

Supplement: Supplementary file 5 [file Image_2.tif]
